# P-2271. Unraveling the Past: Analyzing the Effects of COVID-19 on Hematological Malignancy Patients - Observations from 2020 to 2022

**DOI:** 10.1093/ofid/ofae631.2424

**Published:** 2025-01-29

**Authors:** Francesco Marchesi, Jon Salmanton-Garcia, Francesca Farina, Barbora Weinbergerová, Federico Itri, Julio Dávila-Valls, Sonia Martín-Pérez, Andreas Glenthøj, Ditte Stampe Hersby, Maria Gomes da Silva, Raquel Nunes Rodrigues, Raúl Córdoba, Alberto López-García, Ziad Emarah, Shaimaa El-Ashwah, Yavuz M Bilgin, Laman Rahimli, Oliver A Cornely, Livio Pagano

**Affiliations:** IRCCS Regina Elena National Cancer Institute, Rome, Lazio, Italy; University Hospital Cologne, Cologne, Germany, Cologne, Nordrhein-Westfalen, Germany; IRCCS San Raffaele Scientific Institute, Milan, Lombardia, Italy; Faculty Hospital Brno, Brno, Czech Republic, Brno, Hlavni mesto Praha, Czech Republic; San Luigi Gonzaga Hospital, Orbassano, Piemonte, Italy; Hospital Nuestra Señora de Sonsoles, Ávila, Castilla y Leon, Spain; Hospital Nuestra Señora de Sonsoles, Ávila, Castilla y Leon, Spain; Rigshospitalet, Copenhagen, Hovedstaden, Denmark; Rigshospitalet, Copenhagen, Hovedstaden, Denmark; Portuguese Institute of Oncology, Lisbon, Lisboa, Portugal; Portuguese Institute of Oncology, Lisbon, Lisboa, Portugal; Fundacion Jimenez Diaz University Hospital, Madrid, Madrid, Spain; Fundacion Jimenez Diaz University Hospital, Madrid, Madrid, Spain; Oncology Center Mansoura University, Mansoura, Ad Daqahliyah, Egypt; Oncology Center Mansoura University, Mansoura, Ad Daqahliyah, Egypt; ADRZ, Goes, Zeeland, Netherlands; University Hospital Cologne, Cologne, Nordrhein-Westfalen, Germany; University of Cologne, Faculty of Medicine and University Hospital Cologne, Cologne, Nordrhein-Westfalen, Germany; Fondazione Policlinico Universitario Agostino Gemelli - IRCCS, Rome, Lazio, Italy

## Abstract

**Background:**

During the COVID-19 pandemic, individuals with hematological malignancies faced elevated risks, leading to increased hospitalization and mortality rates, despite the availability of vaccines and treatments for the general population.

**Methods:**

Data on COVID-19 cases in patients with hematological malignancies were collected from diverse global regions. This effort enabled the compilation of extensive epidemiological data for a comprehensive analysis of evolving COVID-19 patterns over three years.

**Results:**

By December 2022, 8,767 COVID-19 cases were reported among hematological malignancy patients from 152 centers in 41 countries, with 42% being female. The distribution included 31% Non-Hodgkin's lymphoma, 17% multiple myeloma, 13% chronic lymphocytic leukemia, and 12% acute myeloid leukemia. Chronic cardiopathy increased from 32% in H1 2020 to 44% in H2 2022. Critical infections dropped from 19% to 4% (p=0.004), with mortality decreasing from 29% to 4% (p< 0.001). Survival rates improved each semester (p< 0.001). Factors linked to mortality included advanced age (HR 1.037, p< 0.001), multiple comorbidities (HR 1.244, p< 0.001), active malignancy at COVID-19 onset (HR 1.832, p< 0.001), pulmonary symptoms (HR 1.299, p=0.003), and hospitalization, especially in the ICU (HR 33.684, p< 0.001). Enhanced survival was associated with anti-SARS-CoV-2 vaccination (1-2 doses: HR 0.681, p< 0.001; 3+ doses: HR 0.451, p< 0.001) and experiencing COVID-19 in 2022 (HR 0.427, p< 0.001). Initially, COVID-19 caused 70% of mortality, decreasing to 42% by H2 2022.

**Conclusion:**

Patients with hematological malignancies remain at heightened risk, despite reductions in critical infections and overall mortality rates. Hospitalization, particularly in ICUs, remains a significant concern. The study highlights the importance of vaccination and the timing of COVID-19 exposure in 2022 for enhancing survival among this group. Ongoing monitoring and targeted interventions are crucial to support this vulnerable population, underscoring the critical role of early diagnosis and swift treatment in mitigating severe COVID-19 cases.

**Disclosures:**

Oliver A. Cornely, Prof. Dr., Abbott: Honoraria|Abbvie: Advisor/Consultant|Abbvie: Honoraria|AiCuris: Advisor/Consultant|Akademie fur Infektionmedizin: Honoraria|Al-Jazeera Pharmaceuticals/Hikma: Honoraria|amedes: Honoraria|AstraZeneca: Honoraria|Basilea: Advisor/Consultant|Biocon: Advisor/Consultant|BMBF: Grant/Research Support|Boston Strategic Partners: Advisor/Consultant|CIdara: Advisor/Consultant|CIdara: Expert Testimony|CIdara: Grant/Research Support|CIdara: Participation on a DRC or DSMB|CoRe Consulting: Stocks/Bonds (Private Company)|Deutscher Arzteverlag: Honoraria|DZIF: Grant/Research Support|EasyRadiology: Stocks/Bonds (Private Company)|EU-DG RTD: Grant/Research Support|F2G: Grant/Research Support|Gilead: Advisor/Consultant|Gilead: Grant/Research Support|Gilead: Honoraria|Grupo Biotoscana/United Medical/Knight: Honoraria|GSK: Advisor/Consultant|GSK: Honoraria|IQVIA: Advisor/Consultant|IQVIA: Participation on a DRC or DSMB|Janssen: Advisor/Consultant|Janssen: Participation on a DRC or DSMB|Matinas: Advisor/Consultant|MedPace: Advisor/Consultant|MedPace: Grant/Research Support|MedPace: Participation on a DRC or DSMB|Medscape/WebMD: Honoraria|MedUpdate: Honoraria|Menarini: Advisor/Consultant|Moderna: Honoraria|Molecular Partners: Advisor/Consultant|MSD: Grant/Research Support|MSD: Honoraria|MSG-ERC: Advisor/Consultant|Mundipharma: Advisor/Consultant|Mundipharma: Grant/Research Support|Mundipharma: Honoraria|Noscendo: Honoraria|Noxxon: Advisor/Consultant|Octapharma: Advisor/Consultant|Octapharma: Grant/Research Support|Pardes: Advisor/Consultant|Partner Therapeutics: Advisor/Consultant|Patent: US18/562644|Paul-Martini-Stiftung: Honoraria|Pfizer: Advisor/Consultant|Pfizer: Grant/Research Support|Pfizer: Honoraria|PSI: Advisor/Consultant|PSI: Participation on a DRC or DSMB|Pulmocide: Participation on a DRC or DSMB|Sandoz: Honoraria|Scynexis: Advisor/Consultant|Scynexis: Grant/Research Support|Seqirus: Advisor/Consultant|Seqirus: Honoraria|Seres: Advisor/Consultant|Shionogi: Advisor/Consultant|Shionogi: Honoraria|streamedup!: Honoraria|The Prime Meridian Group: Advisor/Consultant|Touch Independent: Honoraria|Vitis: Honoraria

